# Dreams and Nightmares during the First and Second Wave of the COVID-19 Infection: A Longitudinal Study

**DOI:** 10.3390/brainsci11111375

**Published:** 2021-10-20

**Authors:** Serena Scarpelli, Valentina Alfonsi, Maurizio Gorgoni, Alessandro Musetti, Maria Filosa, Maria C. Quattropani, Vittorio Lenzo, Elena Vegni, Lidia Borghi, Giorgia Margherita, Maria Francesca Freda, Emanuela Saita, Roberto Cattivelli, Gianluca Castelnuovo, Tommaso Manari, Giuseppe Plazzi, Luigi De Gennaro, Christian Franceschini

**Affiliations:** 1 Department of Psychology, Sapienza University of Rome, 00185 Rome, Italy; valentina.alfonsi@uniroma1.it (V.A.); maurizio.gorgoni@uniroma1.it (M.G.); luigi.degennaro@uniroma1.it (L.D.G.); 2 Department of Humanities, Social Sciences and Cultural Industries, University of Parma, 43125 Parma, Italy; alessandro.musetti@unipr.it (A.M.); tommaso.manari@unipr.it (T.M.); 3 Department of Medicine and Surgery, University of Parma, 43126 Parma, Italy; filosa.maria@hotmail.it (M.F.); christian.franceschini@unipr.it (C.F.); 4 Department of Clinical and Experimental Medicine, University of Messina, 98122 Messina, Italy; mquattropani@unime.it; 5 Department of Social and Educational Sciences of the Mediterranean Area, “Dante Alighieri” University for Foreigners of Reggio Calabria, 89125 Reggio Calabria, Italy; v.lenzo@unidarc.it; 6 Department of Health Sciences, University of Milan, 20146 Milan, Italy; elena.vegni@unimi.it (E.V.); lidia.borghi@unimi.it (L.B.); 7 Department of Humanistic Studies, Federico II University, 80138 Naples, Italy; margheri@unina.it (G.M.); mariafrancesca.freda@unina.it (M.F.F.); 8 Department of Psychology, Catholic University of Milan, 20123 Milan, Italy; emanuela.saita@unicatt.it (E.S.); roberto.cattivelli@unicatt.it (R.C.); gianluca.castelnuovo@unicatt.it (G.C.); 9 Psychology Research Laboratory, Istituto Auxologico Italiano IRCCS, 28824 Verbania, Italy; 10 IRCCS Istituto delle Scienze Neurologiche di Bologna, 40139 Bologna, Italy; giuseppe.plazzi@unibo.it; 11 Department of Biomedical, Metabolic and Neural Sciences, University of Modena and Reggio Emilia, 41125 Modena, Italy; 12 IRCCS Fondazione Santa Lucia, 00179 Rome, Italy

**Keywords:** dreaming, nightmares, lucid dreams, sleep, COVID-19, pandemic, lockdown, second wave

## Abstract

Recent literature shows that the Coronovirus-19 (COVID-19) pandemic has provoked significant changes in dreaming. The current study intends to provide an update about dream variable changes during the second wave of COVID-19. A total of 611 participants completed a web survey from December 2020 to January 2021. Statistical comparisons showed that subjects had lower dream-recall frequency, nightmare frequency, lucid-dream frequency, emotional intensity, and nightmare distress during the second than the first wave of the pandemic. Dreams had a higher negative tone during the second than first wave. We revealed significant differences concerning post-traumatic growth, sleep-related post-traumatic stress disorder (PTSD) symptoms and sleep measures between groups obtained as a function of the changes in the oneiric frequency between the first and second waves. We also found significant correlations between qualitative/emotional dream features and COVID-19-related factors (job change, forced quarantine, having COVID-19 infected relatives/friends, or asking for mental health help). Overall, we found that the second wave affected fewer quantitative features of dream activity and there was less emotional intensity. Moreover, we confirmed the relationship between nightmares and the high risk of PTSD when subjects were grouped as a function of the increasing/decreasing frequency. Finally, our findings are partly coherent with the continuity hypothesis between oneiric and waking experiences.

## 1. Introduction

The 2019 novel coronavirus (COVID-19) affected people’s lives worldwide. On 11 March 2020, the World Health Organization declared COVID-19 a pandemic [1], representing a challenge to all health care systems and governments. Several measures to face and prevent the infection have been adopted, such as social distancing, the obligation to wear masks, and quarantine. The Italian government was the first country in Europe to state a general lockdown to manage the spread of the virus. After the first contagion peak (from March to May 2020), the restrictions were eased until December 2020 [2], when the second wave of infections required new restrictions and a partial lockdown based on the number of infected and hospitalized people in each Italian region [2].

Against this background, a growing number of research papers were showing that the outbreak was associated with relevant psychological symptoms [3,4] and sleep quality was significantly worsened [4,5,6]. Many investigations also explored oneiric activity during the pandemic [7,8,9,10,11,12,13,14,15,16]. Results from different countries revealed higher dream-recall frequency during the pandemic (e.g., [16]). Moreover, subjects reported that dream content was pandemic-related [7,8,9,17]. In particular, studies on Italian samples revealed that poor sleep quality was associated with the high dream-recall frequency [12,15]. Further, the subjects more affected by COVID-19 reported higher emotional intensity in their dreams [12,15,18]. Additionally, stable characteristics, i.e., age and gender modulated the oneiric frequency. Indeed, during lockdown women showed greater self-reported dream frequency [12,13,15], and older adults reported lower dream rates [12,15,17]. In addition, nightmares increased during the Italian lockdown [12]. Both high dream-recall frequency [15] and the nightmare rate [12] were associated with poor sleep quality, depression, and anxiety symptoms, highlighting the relationship between waking emotional experience and the oneiric world [19]. 

It is worth noting that the mentioned investigations were mainly carried out during the lockdown period. Just a few studies aimed to understand the stability of the observed changes in dream activity at different pandemic stages. In this respect, Scarpelli et al. [14] longitudinally investigated the impact of the easing of restrictions on dreaming during the first week after lockdown. The results showed higher dream recall and lucid-dream frequency during lockdown than post-lockdown [14]. Further, people reported greater dreaming, including crowded places when they could go out again after the prolonged confinement. To the best of our knowledge, just the study by Conte and colleagues [17] examined the changes in dreaming both during the lockdown and the second wave. They revealed that significantly fewer participants reported dream changes (frequency, length, and vividness) during the second wave of pandemic, compared with the first. In this view, the authors suggested that people perceived fewer changes in their dreams since their life and daily routine were less impacted by the second, partial, lockdown [17]. Moreover, increased emotional charge was found both during the first and second wave, while subjects reported that dream emotionality returned almost to baseline between the first and second lockdown [17]. However, the study did not provide a direct comparison between dream features of the two waves and very little is known about the dream and nightmare changes across the COVID-19 pandemic.

In light of the current knowledge about dreaming, sleep, and psychological wellbeing during the pandemic, we aimed to provide a within-subject comparison between quantitative and qualitative oneiric variables, considering traditional dreams, lucid dreams, and nightmares, during the first and second wave of the COVID-19 infection. We also investigated the effects of the pandemic and confinement on trauma-related changes (i.e., post-traumatic growth) and sleep measures, as a function of the modifications in the oneiric frequency between the first and the second wave. We expected that the second wave might have a lower impact on oneiric activity than the total lockdown in spring (March-May) 2020. Finally, focusing on the second wave, we aimed to explore the relationships between COVID-19-related variables and the emotional features of dream activity, hypothesizing that COVID-19-related waking experiences can affect the emotional dream/nightmare features.

## 2. Materials and Methods

### 2.1. Participants and Study Design

Subjects who previously completed an online survey developed for the COVID-19 pandemic (spring 2020, first wave) were requested to fill out a new survey on the Microsoft Azure platform during the second wave of the outbreak (18 December–18 January). The survey took approximately 30 min and only participants aged ≥18 years and living in Italy were included in the final sample.

The web survey was promoted on different social media and via university platforms and virtual learning environments. 

Similarly to the survey completed during the first wave of pandemic, subjects filled out a self-administered questionnaire to collect socio-demographic information, psychological and sleep measures, and dream variables. 

Subjects signed the informed electronic consent form before accessing the survey. They were asked to create the same identification code used for the previous version of the survey. Again, participants explicitly agreed to provide an email contact. The protocol was approved by the local ethics committee (CERIP- Comitato Etico del Centro di Ricerca e di Intervento Psicologico—University of Messina, 4 March 2021 prot. n. 12106), according to the Declaration of Helsinki.

A total of 648 subjects who took part in the first round of the web survey (T1) completed the follow-up survey (T2). Thirty-seven subjects were excluded: 33 reported to have been COVID-19 positive and 4 subjects were under 18. The final sample consisted of 611 subjects. Data from healthy subjects reported in the current study have been presented elsewhere [12,14,19,20] and were part of a more comprehensive project, “Resilience and the COVID-19: how to react to perceived stress. Effects on sleep quality and diurnal behavior/thoughts”, with different objectives concerning the impact of lockdown on the Italian population.

### 2.2. Measures

#### 2.2.1. Socio-Demographic and COVID-19-Related Information

We collected the following information: age, gender, marital status, presence/absence of children, education level, Italian area, occupation, job change, job loss, forced quarantine (previous three months), having friends or relatives infected by or dead due to COVID-19 (previous three months), and asking for help from a mental health professional and/or a sleep specialist.

#### 2.2.2. Post-Traumatic Growth Inventory (PTGI)

The Post-Traumatic Growth Inventory (PTGI; [21]) was used to evaluate the psychological changes in the aftermath of a trauma. Subjects were requested to fill out the PTGI according to their psychological changes related to the COVID-19 pandemic. The questionnaire contains 21 items divided into five dimensions: relating to others (PTGI-RO), new possibilities (PTGI-NP), personal strength (PTGI-PS), spiritual change (PTGI-SC), and appreciation of life (PTGI-AL). Each item is scored on a scale of 0 (“never”) to 5 (“a great degree”). The total score of the PTGI is the sum of all item scores. A higher score indicates trauma-related positive psychological changes.

#### 2.2.3. Pittsburgh Sleep Quality Index Addendum (PSQI-A)

To evaluate the presence of trauma-related subjective sleep disturbances, we administered the PSQI-Addendum (PSQI-A; [22]). The PSQI-A is a self-reported questionnaire assessing seven disruptive nocturnal behaviors frequently detected among individuals suffering from post-traumatic stress disorder (PTSD): flashes; general nervousness; memories or nightmares of traumatic experience; severe anxiety or panic not related to traumatic memories; bad dreams not related to traumatic memories; episodes of terror or screaming during sleep without fully awakening; and episodes of acting out dreams, such as kicking, punching, running, or screaming. A PSQI score ≥ 4 represents a reliable cut-off for discriminating between subjects with and without PTSD [22].

#### 2.2.4. Sleep Hygiene Index (SHI)

The SHI [23] is a 13-item self-reported questionnaire to evaluate practices and behaviors related to sleep hygiene. The frequency with which subjects have engaged in a specific behavior was rated on a 5-point Likert scale (“never, “rarely, “sometimes”, “frequently”, and “always”). A total score (range 13–65) is calculated by summing items. Higher scores indicate poor sleep hygiene.

#### 2.2.5. Medical Outcomes Study—Sleep Scale (MOS-SS)

Sleep measures were assessed by the Italian adaptation of the Medical Outcomes Study-sleep scale (MOS-SS) [24], a 12-item self-reported questionnaire evaluating sleep quality and quantity within a month. Ten of the 12 MOS-SS items are scored on a 6-point categorical scale ranging from ‘‘1 = all of the time’’ to ‘‘6 = none of the time’’. The question about the time required to fall asleep uses a 5-point categorical response scale ranging from ‘‘0 to 15 min,’’ to ‘‘more than 60 minutes’’. ‘‘Sleep duration” is reported by respondents as the average number of hours they sleep each night. All domains except sleep duration are converted from 0 to 100, and item 2 is recorded as the average number of hours slept per night (0–24 h). The instrument includes six measures for sleep quality (sleep disturbance, snoring, awakening short of breath or with headache, sleep adequacy, somnolence, and sleep duration/optimal sleep). Specifically, in the current study we considered the following domains: sleep disorder and sleepiness.

#### 2.2.6. Mannheim Dream Questionnaire (MADRE)

Dream activity was assessed by the Italian adaptation of the Mannheim Dream Questionnaire (MADRE) [25], a 20-item self-reported questionnaire. This instrument allowed us to measure (a) dream-recall frequency (item 1) rated by a 7-point scale (0 = never and 6 = almost every morning); (b) emotional intensity of dream contents (item 2) rated by a 5-point scale (0 = not at all intense and 4 = very intense); (c) emotional tone (item 3) rated by a 5-point scale (−2 = very negative and 2 = very positive); (d) nightmare frequency (item 4) rated by an 8-point scale (0 = never and 8 = several times a week); (e) nightmare distress (item 5) rated by a 5-point scale (0 = not at all distressing and 4 = very distressing); and (f) lucid-dream frequency (item 10) rated by an 8-point scale (0 = never and 8 = several times a week). The frequency was asked with reference to the previous month. Further, the instrument assessed individuals’ attitudes towards dreams by item 12 consisting of eight sentences with a 5-point format (0 = not at all and 4 = totally) and the impact of dream on daily life (the frequency of dream sharing, the recording of dreams, the dreams affecting daytime mood, the creative dreams, and the problem-solving dreams) was assessed with items 13 to 17). Dream variables eliciting utilization of dreams (i.e., frequency of dream sharing, recording of dreams, dreams affecting daytime mood, creative dreams, problem-solving dreams) were in an 8-point scale with 0 = never and 8 = several times a week. The questionnaire allowed us to also collect information also on recurring nightmares (items 6 and 7), deja-vu experiences based on dreams (item 18), age of first lucid dream (item 11), reading about dreams (item 19, rated in a 3-point scale), and helpful dream literature (item 20, rated in a 5-point scale).

In the current paper, items investigating state-like features of dream activity (items 1, 2, 3, 4, 5, and 10) were considered for further analysis.

### 2.3. Statistical Analysis

First, descriptive analyses were carried out to outline the sociodemographic features of the sample, considering the following information: age, gender, marital status, presence/absence of children, education level, Italian area, occupation, job change, job loss, forced quarantine (previous three months), having friends or relatives infected by or dead due to COVID-19 (previous three months), and asking for help from a mental health professional and/or a sleep specialist.

With the aim of exploring differences in dreaming between the first (T1) and second (T2) wave of the COVID-19 pandemic, we performed a one-way repeated-measures multivariate analysis of variance (MANOVA), with “time” (T1 versus T2) as the within-subject factor, and quantitative (dream, nightmare, and lucid-dream frequencies) and qualitative emotional features (emotional intensity, emotional tone, and nightmare distress) as dependent variables. To understand which conditions were different at the univariate level, we performed ANOVAs.

Then, focusing on the T2 sample, we considered three different subgroups as a function of the changes in the frequency compared to the T1 phase [increased (+), decreased (−), or not changed (=)] for all of the dream types considered [dream-recall frequency (DRF), nightmare frequency (NF), or lucid-dream frequency (LDF)]. The three groups obtained for each dream category were compared by one-way MANOVA considering the following dependent variables: PTGI-RO, PTGI-NP, PTGI-PS, PTGI-SC, PTGI-AL, PSQI-A, SHI, sleep disorders, and sleepiness. 

To explore the relationship between COVID-19-related variables and qualitative dream features, we performed point-biserial correlations. Specifically, emotional intensity, emotional tone, and nightmare distress were correlated with job change, job loss, forced quarantine (last three months), having friends or relatives infected by or dead due to COVID-19 (last three months), asking for help from a mental health professional and/or a sleep specialist during the second wave.

The statistical analyses were performed using Statistical Package for Social Sciences (SPSS) version 25.0. *p*-values of less than 0.05 were considered statistically significant.

## 3. Results

### 3.1. Characteristics of Samples

The characteristics of participants are shown in Table 1. Briefly, 39.4% of the sample were young subjects (age 18–25). Participants were mostly females (79.1%). Subjects were mainly single (33.9%), but a high percentage of subjects was engaged (27%) or married (23%). Most of the subjects had received a high school education (47%). A high percentage of respondents were employed (49.3 %) or students (43.9%). A small percentage of subjects had lost or changed their job during the second wave (around 8%). In addition, 77.3% came from North Italy. More than 70% of the individuals did not have children. Most of the respondents did not experience forced quarantine (87.2%). Almost half of the sample had COVID-19-infected friends or relatives (45.3%). Finally, more than 90% of the participants did not have close people dead due to the COVID-19 infection. A small percentage of the sample (14.9%) asked for help from a mental health service during the second wave. Finally, 2.1% of participants asked for help from a sleep specialist.

### 3.2. Dream Activity Changes between First and Second Wave of Pandemic

We selected participants from our previous study (T1) [12] who also took part in the second time-interval (T2) and our final longitudinal sample consisted of 605 subjects.

**Figure 1:**
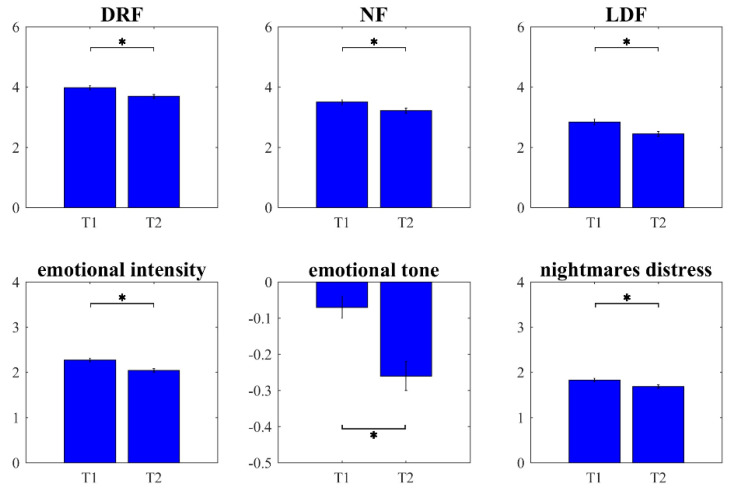
Dream activity changes between first and second wave of pandemic. Results of within-subject comparisons of dream-recall frequency (DRF), nightmare frequency (NF), lucid-dream frequency (LDF), emotional intensity, emotional tone, and nightmare distress of the first (T1) vs. the second (T2) wave. Error bars represent the standard errors. Significant results are asterisked (* *p* < 0.05).

One-way MANOVA comparing the qualitative and quantitative self-reported dream features between the first and second wave of the outbreak showed a statistically significant difference (Wilks’ λ = 0.84, F_6599_ = 18.79, *p* < 0.001, η² = 0.158). Univariate ANOVAs revealed that subjects had lower DRF (F_1604_ = 20.58, *p* < 0.001), NF (F_1604_ = 19.97, *p* < 0.001), and LDF (F_1604_ = 20.24, *p* < 0.001) during the second wave than the first one. Similarly, emotional intensity (F_1604_ = 26.53, *p* < 0.001) and nightmare distress (F_1604_ = 9.81, *p* = 0.002) were significantly lower during the second than the first wave of the pandemic. Dreams were also characterized by a higher negative tone during the second wave than the first one (F_1604_ = 32.36, *p* < 0.001). Significant differences are shown in Figure 1. 

### 3.3. Psychological and Sleep Measures Differ between Groups Based on Recall Frequency

Overall, the groups created as a function of oneiric frequency were composed as follows: “+DRF ” *n* = 161, “−DRF ” *n* = 239, “=DRF ” *n* = 209; “+NF” *n* = 153, “−NF” *n* = 258, “=NF” *n* = 200; “+LDF” *n* = 167, “−LDF” *n* = 233, “=LDF” *n* = 208.

One-way MANOVA comparing self-reported psychological and sleep measures between +DRF vs. −DRF vs. =DRF during the second wave showed no significant effect (Wilks’ λ = 0.97, F_181,196_ = 1.05, *p* = 0.40, η² = 0.016). Conversely, one-way MANOVA comparing psychological and sleep measures between +NF vs. −NF vs. =NF (Wilks’ λ = 0.93, F_181,200_ = 2.55, *p* < 0.001, η² = 0.037) and between +LDF vs. −LDF vs. =LDF (Wilks’ λ = 0.95, F_181,194_ = 1.70, *p* = 0.033, η² = 0.025) showed a significant effect. Specifically, univariate ANOVAs and post-hoc tests (by Bonferroni adjustment) showed that subjects with an increase in NF during T2 had (a) higher PTGI-RO score (*p* = 0.006) than subjects without changes; (b) higher PTSD-related symptoms (PSQI-A score) (*p* < 0.001) than the other two groups; (c) higher sleepiness (*p* = 0.001) than subjects without changes; (d) higher sleep disorders than subjects without changes (*p* < 0.001) or with a decrease (*p* < 0.001); and (e) lower sleep hygiene (*p* = 0.001) than the group without changes. Subjects with a decrease in NF also showed higher sleepiness (*p* = 0.036) than the group without changes.

**Figure 2:**
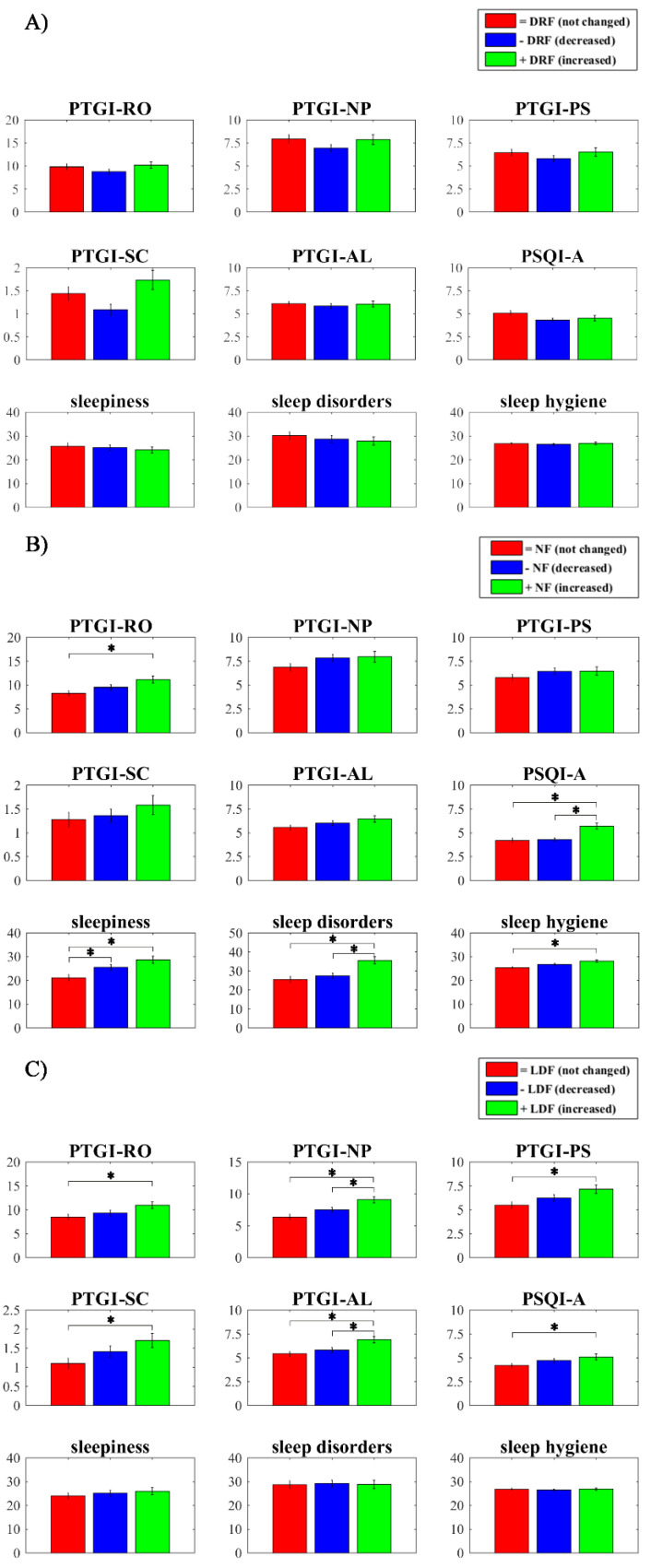
Differences between groups based on recall frequency. (**A**) Comparisons between groups with dream-recall frequency without changes (=DRF; red bars) vs. decreased DRF (−DRF; blue bars) vs. increased DRF (+DRF; green bars) on Post-Traumatic Growth Inventory (PTGI) scores (relating to others (PTGI-RO), new possibilities (PTGI-NP), personal strength (PTGI-PS), spiritual change (PTGI-SC), and appreciation of life (PTGI-AL)), Pittsburgh Sleep Quality Index Addendum (PSQI-A) score, sleepiness, sleep disorders, and Sleep Hygiene Index (SHI) score. (**B**) Comparisons between groups with nightmare frequency without changes (=NF; red bars) vs. decreased NF (−NF; blue bars) vs. increased NF (+NF; green bars) on (PTGI) scores (PTGI-RO, PTGI-NP, PTGI-PS, PTGI-SC, and PTGI-AL), PSQI-A score, sleepiness, sleep disorders, and SHI score. (**C**) Comparisons between groups with lucid-dream frequency without changes (=LDF; red bars) vs. decreased LDF (−LDF; blue bars) vs. increased LDF (+LDF; green bars) on (PTGI) scores (PTGI-RO, PTGI-NP, PTGI-PS, PTGI-SC, and PTGI-AL), PSQI-A score, sleepiness, sleep disorders, and SHI score. Error bars represent the standard errors. Significant results are asterisked (* *p* < 0.05).

Further, univariate ANOVAs and post-hoc tests (by Bonferroni adjustment) showed that subjects with an increase in LDF during T2 had (a) higher PTGI-RO score (*p* = 0.022) than subjects without changes; (b) higher PTGI-NP score than subjects without changes (*p* < 0.001) or with a decrease (*p*= 0.041); (c) higher PTGI-PS (*p* = 0.008) and PTGI-SC (*p* = 0.028) than subjects without changes; (d) higher PTGI-AL score than subjects without changes (*p* = 0.001) or with a decrease (*p* = 0.017); and (e) higher PTSD-related symptoms (PSQI-A) score (*p* = 0.045) than subjects without changes. Significant differences are shown in Figure 2.

### 3.4. COVID-19 Related Factors Correlate with Qualitative Dream Features

Point-biserial correlations between self-reported qualitative dream features and COVID-19-related factors revealed that (a) job change was correlated both with higher emotional intensity of dream (r_pb_ = 0.102; *p* = 0.012) and nightmare distress (r_pb_ = 0.117; *p* = 0.004); (b) forced quarantine was related with negative tone of dream contents (r_pb_ = −0.98; *p* = 0.015); (c) having COVID-19 infected relatives/friends was correlated with negative tone of dream contents (r_pb_ = −0.87; *p* = 0.031); (d) asking for help from a mental health service was associated with higher emotional intensity of dreams (r_pb_ = 0.149; *p* < 0.001), negative tone of dream contents (r_pb_ = −0.84; *p* = 0.039), and higher nightmare distress (r_pb_ = 0.133; *p* = 0.001). Correlations are reported in Table 2.

## 4. Discussion

Our study examined the differences between self-reported quantitative and emotional dreams features of the Italian subjects who participated in the web survey during the first and second pandemic waves. To the best of our knowledge, this is the first longitudinal study that showed the effect of lockdown-related changes on different oneiric phenomena (dreams, nightmares, and lucid dreams) collected by standardized measures in a within-subject design. We found that all quantitative dream variables had lower scores during the second wave than during the lockdown period. In other words, dreams, lucid dreams, and nightmare frequency were reduced during the period between December 2020 and January 2021. Similarly, emotional intensity and nightmare distress were lower during the second wave than during the first wave. These results are consistent with the previous literature on pandemic dreams, confirming that the lockdown period (spring 2020) changed oneiric activity provoking a higher dream production and dreams with greater emotional intensity and nightmare distress [7,12,13,14,15,17]. However, we revealed that during the second wave the emotional valence of dream experience was more negative than during the total lockdown period. Conte et al. [17] also showed that reported dream emotional tone became significantly more negative both in total lockdown and partial lockdown (second wave) compared to previous periods. Moreover, although they did not find a direct correlation between waking negative effect and dream valence, they also found that fear and negative mood during wakefulness were higher during the second than the first wave [17]. Additionally, studies investigating resilience during the pandemic highlighted a reduced capacity of people to adapt well in the second wave’s face [26]. According to this evidence, we can suppose that the increased negative emotional tone of dreams may represent an indicator of this lower resilience [19]. Also, we could hypothesize that even if the general presence of dream activity was reduced in the second wave, it represented a “chronic” condition that negatively influences the mood of dream contents. 

On the one hand, it is worth noting that a complete understanding of the relationship between waking and oneiric emotionality is still lacking. In particular, the processes through waking effects impact on the dream valence deserve further investigations [27,28]. On the other, the restrictions were more relaxed than during the total lockdown, which may imply a different influence on waking experiences. In this respect, we showed that specific COVID-19-related waking conditions were correlated with emotional features of dreaming. Indeed, job change was associated with the greater emotional load of dreams and forced quarantine with the negative tone of dream contents. Moreover, having COVID-19-infected relatives/friends was correlated with the negative tone of dream contents and asking for help from a mental health service was linked with the higher emotional intensity of dreams, the negative tone of dreams, and the higher nightmare distress. These results could be interpreted in light of the continuity hypothesis, which states that the dream scenario reflects the dreamer’s current concerns and psychologically meaningful and salient experiences [29,30]. 

Importantly, we found significant differences concerning self-reported psychological and sleep measures between groups created as a function of the changes in the self-evaluated oneiric frequency between the first and the second wave. Specifically, we revealed that sleep disturbances and sleepiness, as well as lower sleep hygiene, characterized the group with increased NF during T2. It is not surprising that people with more nightmares had higher sleep problems and poor sleep hygiene, as highlighted by the previous literature [31,32]. Specifically, the research on trauma (e.g., [33,34]) and pandemic dreaming (e.g., [12,14]) underlined that disrupted sleep and nightmares might occur in parallel following a traumatic event. Even if we did not collect nightmares’ contents, we may hypothesize that the group with higher NF may be considered at high risk of developing PTSD [35] since the pandemic likely qualified as a traumatic event. Accordingly, the current literature on the COVID-19 pandemic highlighted that all changes during wakefulness due to the confinement, restrictions, and the fear of infection might be considered as adverse events increasing the risk of PTSD [36]. 

Additionally, all PTGI dimensions (relating to other, new possibilities, personal strength, spiritual change, and appreciation of life) were increased in the LDF+ group. In parallel, PTSD-sleep-related symptoms measured by PSQI-A were greater in the LDF+ group than the group without changes. Similarly, subjects with higher NF in T2 than T1 showed higher post-traumatic growth concerning relations with others and greater PTSD-related symptoms as well. Namely, we found that subjects with increased NF and LDF during T2 had—at the same time—both higher scores of PTGI and PSQI-A. It should be noted that the existing evidence on the relationship between post-traumatic growth (the benefit-finding after adverse events) and PTSD symptoms is mixed [37]. Our findings are consistent with studies showing a coexistence between growth and distress after trauma exposure (e.g., [38]). More directly, a recent investigation on discharged COVID-19 patients revealed that post-traumatic growth was positively correlated with PTSD symptoms [39]. Accordingly, our results are coherent with the view of a quadratic (inverted-U) relationship between post-traumatic growth and PTSD [37]. Further, we can speculate that PTSD-related symptoms and post-traumatic growth are not necessarily opposite phenomena, but more likely complementary, especially at an early stage after a traumatic event. In other words, even if individuals suffer from high psychological distress, they could identify some positive post-trauma changes, such as the possibility of dealing with even the most severe of challenges, redefining personal strengths and relationships in their lives [39,40]. In this view, the fact that individuals with increased LDF had higher scores in all PTGI dimensions is quite consistent with the hypothesis that lucid dreaming could play some role in emotional regulation processes in people experiencing adverse events [41]. Along this vein, previous studies showed that lucid dreaming was greater during lockdown than in the post-lockdown week [14]. However, we have to emphasize that the exact development of the relationship between post-traumatic growth, PTSD symptoms, and dreaming is still unknown and deserves further investigation. 

Overall, our findings are consistent with the idea that dreaming may be a reliable index of individuals’ well-being. Considering the re-organization of the healthcare system to tackle the COVID-19 pandemic (e.g., [42,43]), we suggest that investigating nightmares and dreaming could help the management of the psychological consequences of the outbreak.

## 5. Conclusions

We highlighted that the reported oneiric frequency and emotional intensity of dreams were globally reduced during the second compared to the first wave. More importantly, we confirmed the relationship between nightmares and psychological discomfort/high risk of PTSD when subjects were grouped as a function of the increasing/decreasing frequency. Our findings also provided support for the continuity hypothesis [29].

The current study had some limitations. First, our data were retrospectively collected by a self-reported questionnaire on the web. We are aware that this method could affect the collection of dream activity since it is not exempt from memory bias [44]. Second, more than half of our sample was composed of women. Even if this issue concerned several web survey pandemic studies (e.g., [45,46]), we must underline that our results should be taken with caution and are not generalizable to the whole Italian population. Third, due to the longitudinal design of this study, we had relevant dropout rates after the initial recruitment. Finally, we did not have direct information on PTSD symptoms. In fact, the PSQI-A allowed us to collect only sleep-related PTSD symptoms. Further, we could not perform within-subject comparisons on PTSD-related symptoms between the first and second wave since we did not collect data from PSQI-A during spring 2020. To better understand the relationship between PTSD and post-traumatic growth, more specific measures should be considered. 

To sum up, this study represents an additional step to understanding the meaning of dream changes during the pandemic. Nevertheless, further investigation should be carried out to identify potential confounding and/or mediating factors (e.g., financial burden and household income) influencing oneiric activity across different pandemic stages.

## Figures and Tables

**Table 1:** Characteristics of the sample.

	Participants (*n* = 611) *n* (%)
Age	
18–25	241 (39.4)
26–30	109 (17.8)
31–40	87 (14.2)
41–50	77 (12.6)
51–60	77 (12.6)
60+	20 (3.3)
Gender	
Male	128 (20.9)
Female	483 (79.1)
Marital status	
Single	207 (33.9)
Married	141 (23.0)
Cohabitating	74 (12.1)
Engaged	165 (27.0)
Divorced/separated/widow/widower	24 (4.0)
Education level	
Until middle School	10 (1.6)
High school	287 (47.0)
Bachelor’s degree	114 (18.7)
Master’s degree	146 (23.9)
PhD/postgraduate school	54 (8.8)
Occupation	
Retired	9 (1.5)
Unemployed	33 (5.4)
Student	268 (43.9)
Employed	301 (49.3)
Job change	
Yes	50 (8.2)
No	561 (91.8)
Job loss	
Yes	49 (8)
No	562 (92)
Italian area ^a^	
North Italy	471 (77.3)
Centre and South Italy	138 (22.7)
Having children	
Yes	157 (25.7)
No	454 (74.3)
Forced quarantine	
Yes	78 (12.8)
No	533 (87.2)
COVID-19-infected friends/relatives	
Yes	277 (45.3)
No	334 (54.7)
Friends/relatives dead due to COVID-19	
Yes	54 (8.8)
No	557 (91.2)
Asking for help from a mental health professional	
Yes	91 (14.9)
No	520 (85.1)
Asking for help from a sleep specialist	
Yes	12 (2.1)
No	598 (97.9)

^a^ *n* = 609.

**Table 2:** Correlations between qualitative dream features and COVID-19-related factors (*n* = 611).

	Emotional Intensity	Emotional Tone	Nightmare Distress
**Job loss**	0.073 (0.070)	−0.068 (0.095)	0.076 (0.060)
**Job change**	**0.102 (0.012)**	−0.065 (0.108)	**0.117 (0.004)**
**Forced quarantine**	0.055 (0.176)	−0.098 (0.015)	0.058 (0.153)
**COVID-19 infected relative/friends**	0.070 (0.085)	**−0.087 (0.031)**	0.022 (0.590)
**Relatives/friends dead due to COVID-19**	0.035 (0.388)	−0.032 (0.433)	−0.011 (0.785)
**Asking for help from a mental health professional**	**0.149 (<0.001)**	**−0.084 (0.039)**	**0.133 (0.001)**
**Asking for help from a sleep specialist**	0.029 (0.480)	−0.010 (0.805)	0.044 (0.280)

Significant results are in bold.

## Data Availability

The data presented in this study are available on request from the corresponding author.
